# Feasibility of online managing cancer and living meaningfully (CALM) in Chinese patients with metastatic breast cancer: a pilot randomized control trial

**DOI:** 10.1038/s41598-024-52574-7

**Published:** 2024-02-28

**Authors:** Yening Zhang, Ying Pang, Yi He, Miaoning You, Lili Tang

**Affiliations:** 1 Key Laboratory of Carcinogenesis and Translational Research (Ministry of Education/Beijing), Department of Psycho-Oncology, Peking University Cancer Hospital and Institute, Fu-Cheng Road 52, Hai-Dian District, Beijing, 100142 China; 2 Key Laboratory of Carcinogenesis and Translational Research (Ministry of Education/Beijing), Department of Bresat Oncology, Peking University Cancer Hospital & Institute, Beijing, China

**Keywords:** Palliative care, Breast cancer, Anxiety, Depression, Cancer, Psychology

## Abstract

Metastatic breast cancer could cause various psychological symptoms. Managing Cancer and Living Meaningfully (CALM) is a brief, manualized psychotherapy that has been validated for advanced cancer patients. We conducted a pilot randomized control trial (RCT) to verify the feasibility and preliminary efficacy of CALM therapy in this population. Patients who met the inclusion criteria were randomly assigned into CALM or Wait-list Control (WLC) groups. Patients in the CALM group received CALM therapy and usual care; patients in WLC group first received usual care and then underwent CALM therapy after completing all assessments. All patients were asked to complete three assessments: T0(baseline), T1(3 months), and T2(6 months). The primary outcomes was death anxiety; other outcomes were depression, distress, suicide ideation, attachment security, spiritual well-being and quality of life at the end of life. Analysis of Covariance (ANCOVA) and t-test were used for statistics analysis. Thirty-six patients were randomly assigned to either of the two groups, with 34 patients completing the three assessments. At six months, we found significant between group differences in suicide ideation, distress, and life completion between the CALM and WLC groups. At T2, patients in CALM group reported lower levels of depression (F = 5.016, *p* = 0.033, partial η^2^ = 0.143), distress (F = 7.969, *p* = 0.010, partial η^2^ = 0.257), attachment avoidance (F = 4.407, *p* = 0.044, partial η^2^ = 0.128), and better sense of life completion (F = 5.493, *p* = 0.026, partial η^2^ = 0.155) than patients in the WLC group. Compared with results of the T0 assessments, we found significant differences in socres for depression (T2&T0, t = − 2.689, *p* = 0.011, Cohen’s d = 0.940) and distress (T2&T0, t = − 2.453, *p* = 0.022, Cohen’s d = 0.965) between the two groups. CALM therapy was well received by the study population, and CALM therapy can reduce depression, distress, attachment avoidance while improving quality of life in Chinese metastatic breast cancer patients. A Phase III RCT was recommended to verify the impact of CALM therapy on psychological burden and survival in this population.

*Trial registration*: This study is part of the “Preliminary application study for Managing Cancer and Living Meaningfully (CALM) therapy in Chinese advanced cancer patients” clinical trial, with the Trial Registration Number of ChiCTR1900023129 (13/05/2019) in the Chinese Clinical Trial Registry (ChiCTR) website. (https://www.chictr.org.cn/index.html).

## Introduction

The latest cancer statistics from the National Cancer Center in China indicate that breast cancer remains the most common type of cancer among women in terms of incidence (45.37‰), while the mortality rate (10.62‰) ranked fifth after lung, stomach, liver, and colorectal cancers^1^. With increased national medical resources, the 5-year survival rate of cancer in China has improved significantly from 30.9% in 2003 to 40.5% in 2015, and the 5-year survival rate for breast cancer has increased from 73.1 to 82.0%^2^. Nevertheless, approximately 3%-10% of new breast cancer cases each year still present remote metastases at initial diagnosis. In addtion, 30% of early stages patients eventually progress to advanced stages; and the five-year survival rate for this population is only 20%^3^. When breast cancer progresses beyond cure, the quality and duration of patient' survival becomes even more challenging; disease invasion and clinical treatment produce more somatic symptoms, such as pain and fatigue, and patients' quality of life diminishes significantly^4^; In additon, the psychological burden also increases, and there is a preponderance of anxiety and depression, which interact with physical symptoms^5^. Several studies has demonstrated that 84.1% of patients with advanced breast cancer have clinical anxiety and 25.2% have clinical depression^6^. The impact of depression is particularly severe in patients with advanced disease. The findings of previous studies have revealed that depression can affect patient survival by decreasing the patient’s ability to care for themselves, thus decreasing patient compliance with antitumor treatment^7^.

Fear of death is a fundamental aspect of human existence, and this fear is amplified when an individual become truely aware of inevitability of death. Death anxiety is a prevalent psychosocial issue among patients with advanced cancer. Several studies have demonstrated that 32% of advanced cancer patients experience significant levels of death anxiety^8^ while 43% of advanced non-small cell lung cancer patients experience such level of death anxiety^9^. A domestic study of Chinese patients reports that 37.6% of young women with advanced breast cancer experience considerable death anxiety^10^. Factors influencing death anxiety in patients with advanced cancer include depression, demoralization, fear of disease progression, and declining intimacy^11^. Hence, high-quality psychological interventions for patients with advanced breast cancer should address death anxiety and related factors. Terror Management theory proposes that cultivating a sense of personal worth (a feeling that one's life has meaning and value) is a protective factor for death anxiety and recommends its inclusion in death anxiety interventions^12^.

Focusing on psychosocial issues in patients with advanced cancer and providing positive and effective psychosocial support has become an important part of the guidelines for quality cancer care in several countries and is strongly recommended by some professional organizations. The Breast Health Global Initiative has developed supportive and palliative care programs for low and middle income countries that include systematic psychosocial and spiritual care^13^. The National Comprehensive Cancer Network (NCCN) palliative care guidelines recommend the early integration of palliative care into oncology care to improve quality of life for patients with advanced breast cancer^14^. Notably, Chinese expert consensus on the clinical diagnosis and treatment of advanced breast cancer emphasized multidisciplinary professional care-including psychosocial care^15^. Kissane et al.^16^ verified that supportive expressive group therapy (SEGT) can actively improve depression, hopelessness, trauma symptoms and social functions in patients with metastatic breast cancer. However, group therapy challenges, such as high dropout rates, limit its clinical application in patients with advanced cancer. Although individual cognitive therapy has been trialled in patients with metastatic breast cancer, subsequent researches has not verified its efficacy^17^. Managing Cancer and Living Meaningfully (CALM) is an individual, structured psychological intervention for patients with advanced cancer, with a focus on death anxiety and mood adjustment. Continuous studies have been conducted on CALM therapy in Canada and other countries. The findings of a randomized controlled study with a large sample of advanced cancer patients demonstrate that CALM therapy is significantly effective in improving depression and psychological preparation for the end of life^18^. Although CALM therapy has been introduced to China for some years and some of its elements has been applied to psychological therapy for advanced cancer patients, online CALM delivery has not been explored prior to this study. Online psychotherapies has demonstrated feasibility for cancer patients^19^; However, most online psychotherapy focuse on cognitive and behavior therapy and mindfulness approaches with fixed intervention structures^20,21^. CALM has a relatively flexible structure, and the presentation of the therapeutic domains varies depending on the therapists. No research and clinical application of online CALM therapy has been reported. The COVID-19 pandemic and Community grid management in China limited the feasibility of hospital visits for patients with cancer. Online CALM therapy appeared to be the best option during this period.

Therefore, we conducted this study to explore the following: (1) the feasibility and preliminary efficacy of online CALM therapy for patients with metastatic breast cancer in China; (2) the degree of change that online CALM therapy elicits in this population, as captured by psychometric indicators. 

## Methods

### Patients recruitment

All the patients were recruited from Peking University Cancer Hospital-including outpatients of psycho-oncology department and inpatients at the breast oncology department-between January 2022 to March 2023. This study is part of “Preliminary application study for Managing Cancer and Living Meaningfully (CALM) therapy in Chinese advanced cancer patients (ID: ChiCTR1900023129)” clincal trial, which was registered on the Chinese Clinical Trial Registry (ChiCTR) on 13th May, 2019. This study received ethics approved by Peking University Cancer Hospital Ethics Committee ( No.2019YJZ23). The inclusion criteria were as follows: (1) age ≥ 18 years old; (2) pathologically diagnosed with metastatic breast cancer (UICC TNM stage IV); (3) a ≥15 score on the Death and Dying Distress Scale (DADDS)^22^; (4) capable of understanding questionnaires; (5) able to provide informed consent. Patients with severe cognitive dysfunction and those undergoing systematic psychosocial interventions were excluded. If the enrolled patients feel uncomfortable with the intervention, they were free to stop the intervention or speak to the therapist without any influence on their clinical cancer treatment.

### Study procedure

**Figure 1:**
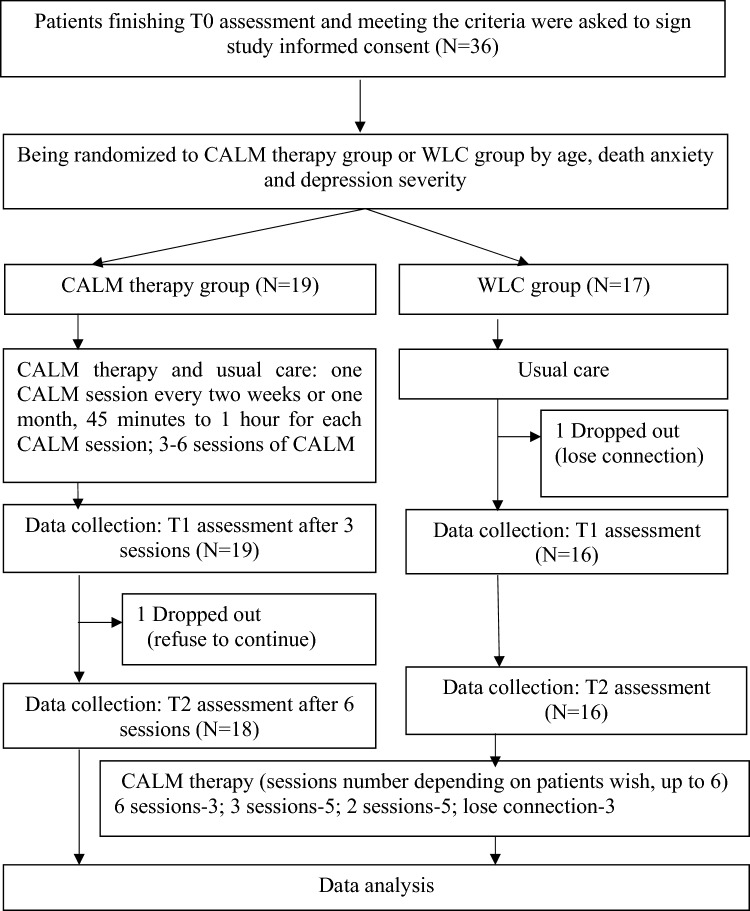
Flowchart in this study.

Patients who met the inclusion criteria were asked to register for recruitment: Step 1: Randomised assignment to the CALM group or the wait-list control (WLC) group—the latter involved receiving usual care first and then subsequently receiving CALM therapy or some other complementary psychosocial care after completing several assessments; Step 2: Receiving CALM therapy (CALM group) or first receiving usual care and then waiting to receive psychosocial care later; Step 3: Undergoing T1 and T2 assessments. The detailed study procedure is outlined in Fig. 1.

The study participants were assigned to the CALM group or the WLC group in a 1:1 ratio. Dynamic Randomization was computer generated centrally by the Peking University Cancer Hospital Clinical Trials Service Unit: Interactive Web Response System. The randomization was stratified to ensure the study sample was representative of the patients population under study powered by age (≥ 45 or otherwise), PHQ-9 score (≥ 10 or otherwise) and DADDS scores (≥ 45 or otherwise), as these indicators have demonstrated substantial prognostic value and correlation with the outcomes or being the primary outcome itself^8,9^. This single-centre, prospective, parallel-controlled clinical study was not blinded, as both patients and researchers were aware of the treatment assignment. Patients in CALM group received CALM therapy along with usual care from oncologists and nurses in the breast cancer department as soon as they were recruited to this study. The WLC group initially received only usual care; however, the participants in the WLC group received CALM therapy following their T2 assessment.

As a pilot trial, the key objectives were to examine the feasibility of implementing the online CALM therapy in Chinese patients with metastatic breast cancer, while also allowing pilot test of potential treatment effects. Thus, a smaller sample size was selected similar as a former pilot study^23^. Mean difference (D) and standard deviation (SD) of efficacy evaluation results were often used as parameters for sample size calculations in randomized controlled studies. A standard deviation (SD) of 2 of the nine-item Patient Health Questionnaire (PHQ-9) was use for sample size calculation in our study. To achieve 80% power for detecting a difference at a 0.05 significance level, sample size calculation using PASS 11.0 software indicated a minimum sample size of 18 for each group. Research coordinators performed the recruitment and group assignment, while a therapist delivered the CALM therapy.

CALM therapy is a brief, structured, individual psychological therapy. CALM therapy focuses on four therapeutic areas: (1) symptom management and communication with medical oncology staff; (2) self-change and relationships with loved ones; (3) sense of meaning and purpose for living; (4) the future and mortality (future, hope, and death). We have added the fifth therapeutic area-(5) fear of cancer progression as the topic was repeated by the patients in the pre-experiment. 3–6 sessions would be carried out in CALM therapy, and 40–60 min for each session. The CALM therapy comprised three to six sessions, with each session lasting 40 to 60 min. Patients in the CALM group received three to six sessions of CALM therapy, along with usual care after T0 assessment, while patients in the WLC group received CALM therapy only after completing theri T1 and T2 assessments. In each CALM session, a brief structure was set out to facilitate the delivery of the therapy by the therapist. The therapy session began with a 10–15 min check-in to assess the patient’s mood and experiences over the past few days. The therapists offered empathetic listening and validation during this segment. For the next 25–40 min, the focus shifted to the core therapeutic areas addressed in CALM therapy. During the last 5 min, therapists worked with patients to summarise the main contents of their discussion during the therapy session and schedule the next session. When this study commenced, COVID-19 restrictions hampered in-person visits to the hospital from which patients were recruited. Therefore, we provided the CALM therapy online via WeChat video calls. The WeChat APP is the most popular online social media platform in China. A previous study has demonstrated the feasibility of WeChat platform-based interventions for patient- education and complementary medical care^24^. We made some adaptations to enable successful delivery online: (1) before the therapy session began, patients were asked to review and verbally consent to the treatment environment (including the room lighting, privacy protections, and surrounding area) in the therapist’s video chat frame, as seen via the webcam. Only after patients consented would the session begin. (2) The therapist checked whether patients felt comfortable in the remote setting and monitored their engagement in the sessions more frequently. (3) Without assess to body language during online therapy, the therapist focused more intently on empathetic verbal communication, emotional recognition and providing support through thoughtful words and listening responses. The therapist in this study is certified in CAML therapy by the Global Institute of Psychosocial Palliative &End-of-Life Care (GIPPEC). The study participants could stop at any time if they were feeling unwell-with the reasons documented. Data from participants who at least completed the T1 assessment were included in the study analysis.

Originally, we aimed to recruit patients with metastatic triple-negative breast cancer as participants in this study. However, the pre-experiment planning at the single center revealed challenges in recruiting a sufficient number of such patients, as the triple-negative breast cancer cases comprise a small proportion of metastatic breast cancer patients. Therefore, recruitment eligibility was broadened to include all metastatic breast cancer subtypes.

### Measurements

#### Main outcome measurements

*Death and Dying Distress Scale (DADDS)*: The 15-item DADDS assesses distress about death and dying in advanced cancer patients^22^. Each item on the instrument is scored on a scale of 0 to 5 points. As recommended by Neel et al., a cut-off point of 45 was used to define death anxiety^8^. The Chinese version of the DADDS has been validated for use with patients with advanced cancer^25^.

#### Other outcome measurements

*Nine item-patient health questionnaires (PHQ-9)*: The PHQ-9 is a validated scale for assessing depression symptoms in patients and is based on the criteria for depression disorder outlined in the Diagnostic and Statistical Manual of Mental Disorders IV (DSM-IV). The Chinese version of the PHQ-9 has demonstrated good validity and reliability among general hospital patients. A ≥10 score was considered indicative of moderate depression^26^.

*Distress Thermometer (DT)*: The single-item DT is recommended by the distress management group of the NCCN. A score of ≥4 is recommended as the cut-off point for significant distress by both the NCCN and a Chinese validation study^27^.

*Functional Assessment of Chronic Illness Therapy-Spiritual Well-Being (FACIT-sp)* This tool was obtained via the FACIT.org website, with a licence to use the simplified Chinese version of the FACIT-Sp in this study. This 12-item scale assesses patients’ spiritual well-being using two subscales: Meaning/Peace (Items 1–8) and Faith (Items 9–12). Each item is scored along a continuum of 0 to 4 (Item 4 and Item 8 are reverse scored), with higher scores indicating better spiritual well-being than lower scores^28^.

*Quality of Life at the End of Life-Cancer (QUAL-EC)*: The 17-item QUAL-EC assesses quality of life at the end of life in advanced cancer patients, with each item scored on a scale of 1 to 5 points^29^. The QUAL-EC comprises four subscales: (1) Symptom Burden (Items 1–3), on which higher scores indicate a greater symptom burden than lower scores; (2) Relationship with Healthcare Provider (Items 4–8), on which high scores reflect better outcomes than low scores; (3) Preparation for End of Life (Items 9–12), on which low scores reflect better outcomes than high scores; and (4) Life Completion (Items 13–17), on which high scores reflect better outcomes than low scores.

*Modified experiences in close relationship scale (ECR-M16)*: The 16-item ECR-M16 is a self-reported measure of attachment security with close others. Two of its subscales are calculated separately. Avoidant attachment is assessed by all the odd-numbered items (Items 3, 7, 11 and 15 are reverse scored). Anxiety attachment is assessed by all the even-numbered items. High scores indicate worse outcomes than low scores on both subscales^30^.

*Clinical Evaluation Questionnaire-2 (CEQ-2)*: The 13-item CEQ-2 elicits patients’ feedback on the feasibility of CALM therapy, covering four CALM domains. Each item is scored on a scale of 0 (not applicable) to 4 (very helpful).

### Statistical analysis

The T-test or a non-parametric test was used to compare the results of the measured psychometric indicators for the CALM group and the WLC group at three time points. The T-test was also used to compare the difference between the CALM group and the WLC group in terms of changes in the measured psychometric indicators, with the effect size denoted by the Cohen’s d value. Analysis of covariance (ANCOVA) was used to compare the post-intervention means of the two groups after adjusting for differences at baseline, which can effectively isolate the effect of the CALM intervention on post-intervention scores and report the effect size using the value of partial η2. All statistical analyses were conducted using the IBM Statistical Package for the Social Sciences (SPSS) 26.0.

### Ethical approval

The cross-sectional study and this intervention study were approved by the Ethics Committees of Peking University Cancer Hospital (No.2018YJZ24 for cross-sectional study and No.2019YJZ23 for the intervention study). All methods were performed in accordance with the guideline of clinic trials required by the Ethics Committees of Peking University Cancer Hospital.

### Consent to participate

We got written informed consent from all participants.

## Results

### Results of demographic information in the sample

Thirty-six participants consented to participate in this intervention study and were randomly assigned to one of two study groups, with 19 participants assigned to the CALM group and 17 assigned to the WLC group (Fig. 1). Data on the participants who received more than three sessions of CALM therapy and/or at least completed the T0 and T1 assessments were included in the statistical analysis. One participant in the CALM group dropped out after three therapy sessions because of the following notion: ‘thinking about I get well and don’t need psychotherapy’. One participant in the WLC group dropped out after the T0 assessment by refusing to answer the call. The mean age of the participants was 47.26 ± 10.032; most of the participants were unreligious, married, city dwellers and medically insured. We found no between-group differences in any of the demographic characteristics (Table 1).

**Table 1:** Differences of demographic information between CALM group and WLC group.

Variables	Total (N = 36)	WLC Group (n = 17) M ± SD/n (%)	CALM Group (n = 19) M ± SD/n (%)	*t /χ*^2^	*p*
Age	47.26 ± 10.032	49.65 ± 10.943	45.11 ± 8.888	1.373	0.179
18–44 years old	15 (41.7)	6 (35.3)	9 (47.4)	0.538	0.463
≥ 45 years old	21 (58.3)	11 (64.7)	10 (52.6)		
Religion				1.092	0.296
No	30 (83.3)	13 (76.5)	17 (89.5)		
Yes	6 (16.7)	4 (23.5)	2 (33.3)		
Marital status				2.043	0.153
Without partner	7 (19.4)	5 (29.4)	2 (10.5)		
Married	29 (80.6)	12 (70.6)	17 (89.5)		
Education status				1.761	0.415
≤ junior middle school	6 (16.7)	2 (11.8)	4 (21.1)		
Senior middle school and junior college	15 (41.7)	9 (52.9)	6 (31.6)		
≥ college	15 (41.7)	6 (35.3)	9 (47.4)		
Living condition				0.037	0.847
In the city	27 (75.0)	13 (76.5)	14 (73.7)		
In rural area	9 (25.0)	4 (23.5)	5 (26.3)		
Average income				2.386	0.303
≤ 3000 RMB/month	8 (22.2)	5 (29.4)	3 (15.8)		
3000–5000RMB/month	15 (41.7)	8 (47.1)	7 (36.8)		
≥ 5000RMB/month	13 (36.1)	4 (23.5)	9 (47.4)		
Payment insurance				0.122	0.727
Self-pay	5 (41.7)	2 (11.8)	3 (15.8)		
By insurance	31 (58.3)	15 (88.2)	16 (84.2)		

***p < 0.001; **p < 0.01; *p < 0.05.

### Primary outcomes

**Figure 2:**
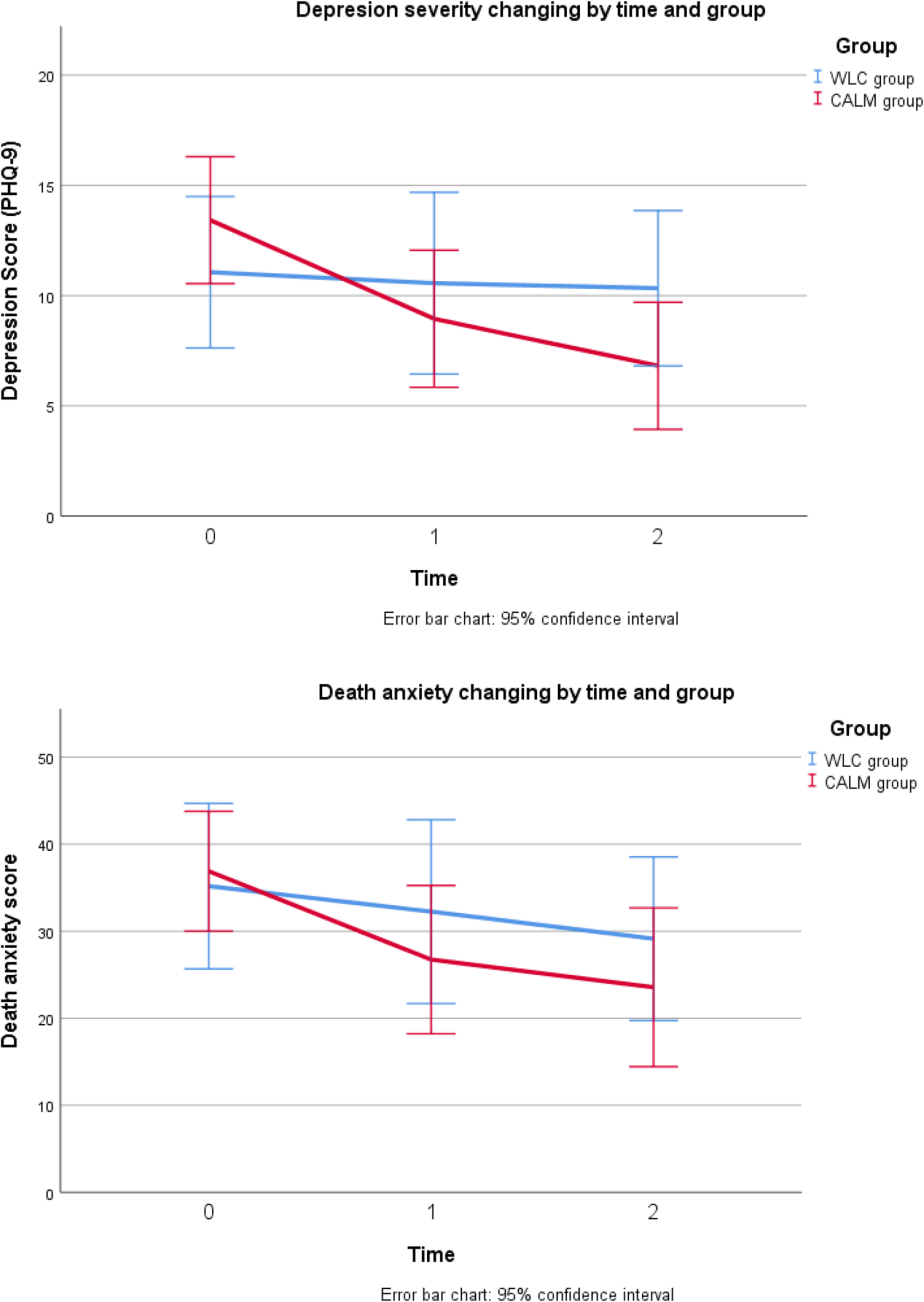
Changing of depression and death anxiety scores in the three time points.

(Table 2), Table 3 presents the primary outcome derived using an analysis of covariance (ANCOVA). For DADDS as primary outcome, no significant between-group differences were found at any time point. However, we found disparities in the death anxiety scores, with better outcomes recorded in the CALM group than in the WLC group; the difference was not significant (T1: 26.74 ± 17.672 vs. 32.25 ± 19.814, *p* = 0.228, partial η2 = 0.045; T2: 23.61 ± 16.256 vs. 29.13 ± 16.974, *p* = 0.271, partial η2 = 0.039). The changing trend is presented in Fig. 2. In addition, the CALM group recorded a considerably large improvement in the primary DADDS outcome (Table 4). However, the results of t-test indicate this within group change is not statistically significant when compared to that in the WLC group (T0-T1: 10.16 ± 13.263 vs. 2.44 ± 21.420, *p* = 0.222, Cohen’s d = 0.442; T0-T2: 13.28 ± 14.652 vs. 5.40 ± 24.994, *p* = 0.268, Cohen’s d = 0.432).

**Table 2:** Differences of measurements indicators between CALM group and WLC group in the three times.

	T0 M ± SD/n (%)		T1 M ± SD/n (%)		T2 M ± SD/n (%)	
PHQ-9 (WLC)	11.06 ± 6.685		9.63 ± 7.145		10.33 ± 6.366	
PHQ-9 (CALM)	13.42 ± 5.957		8.95 ± 6.450		6.82 ± 5.235	
*t*/*χ*^2^	− 1.120		0.295		1.711	
*p*	0.271		0.770		0.097	
Suicide Ideation (WLC)	Negative 11 (64.7)	Positive 6 (35.3)	Negative 8 (50.0)	Positive 8 (50.0)	Negative 6 (40.0)	Positive 9 (60.0)
Suicide Ideation (CALM)	Negative 10 (52.6)	Positive 9 (47.4)	Negative 14 (73.7)	Positive 5 (26.3)	Negative 13 (76.5)	Positive 4 (23.5)
* t*/*χ*^2^	0.538		2.087		4.394	
*p*	0.463		0.149		0.036*	
DADDS (WLC)	35.18 ± 18.484		32.25 ± 19.814		29.13 ± 16.974	
DADDS (CALM)	36.89 ± 14.282		26.74 ± 17.672		23.18 ± 16.648	
*t/χ*^2^	− 0.314		0.870		1.001	
*p*	0.755		0.391		0.326	
DT(WLC)	3.88 ± 2.421		3.00 ± 2.733		4.58 ± 2.644	
DT (CALM)	4.17 ± 2.203		2.42 ± 2.434		2.23 ± 2.127	
*t/χ*^2^	− 0.364		0.663		2.460	
*p*	0.718		0.512		0.022*	
FACIT (WLC)	25.00 ± 10.892		23.13 ± 12.371		27.00 ± 12.364	
FACIT (CALM)	24.84 ± 9.376		27.53 ± 8.322		32.29 ± 7.819	
*t/χ*^2^	0.047		− 1.252		− 1.364	
*p*	0.963		0.219		0.184	
ECR-Avoidant (WLC)	27.88 ± 6.051		29.88 ± 7.527		27.00 ± 12.364	
ECR-Avoidant (CALM)	29.00 ± 6.280		28.11 ± 5.415		32.29 ± 7.819	
*t/χ*^2^	− 0.542		0.807		1.469	
*p*	0.591		0.425		0.152	
ECR-Anxiety (WLC)	26.71 ± 10.999		27.13 ± 11.407		24.80 ± 11.912	
ECR-Anxiety (CALM)	31.26 ± 8.366		28.42 ± 12.025		25.24 ± 11.206	
*t/χ*^2^	− 1.408		− 0.325		− 0.106	
*p*	0.168		0.747		0.916	
QUAL-EC-Relationship (WLC)	17.65 ± 3.605		17.44 ± 3.881		16.00 ± 4.408	
QUAL-EC-Relationship (CALM)	16.63 ± 3.715		15.26 ± 4.026		17.29 ± 4.883	
*t/χ*^2^	0.830		1.618		− 0.783	
*p*	0.412		0.115		0.440	
QUAL-EC-Preparation (WLC)	13.82 ± 3.107		14.38 ± 4.515		13.13 ± 3.114	
QUAL-EC- Preparation (CALM)	14.11 ± 2.885		13.16 ± 4.086		13.29 ± 3.236	
*t/χ*^2^	− 0.282		0.837		− 0.143	
*p*	0.780		0.409		0.887	
QUAL-EC-Life Completion (WLC)	18.06 ± 4.630		17.31 ± 3.877		16.13 ± 3.852	
QUAL-EC-Life Completion (CALM)	17.68 ± 3.449		17.63 ± 3.022		18.71 ± 3.098	
*t/χ*^2^	0.277		− 0.274		− 2.093	
*p*	0.783		0.786		0.045*	

***p < 0.001; **p < 0.01; *p < 0.05.

Significant values are in bold.

**Table 3:** Result from Analysis of Covariance (ANCOVA) in primary and secondary outcomes.

	WLC group	CALM group	F	sig	Effect size from partial Eta Squared (η^2^)
Primary outcome					
DADDS					
T0	34.69 ± 18.976	36.89 ± 14.282	–	–	–
T1	32.25 ± 19.814	26.74 ± 17.672	1.511	0.228	0.045
T2	29.13 ± 16.974	23.61 ± 16.256	1.206	0.271	0.039
Secondary outcomes					
PHQ-9					
T0	11.00 ± 6.899	13.42 ± 5.975	–	–	–
T1	9.62 ± 7.145	8.95 ± 6.450	0.565	0.458	0.017
T2	**10.33 ± 6.366**	**6.94 ± 5.104**	**5.016**	0.033*	**0.143**
DT					
T0	3.88 ± 2.421	4.17 ± 2.203	–	–	–
T1	3.00 ± 2.733	2.42 ± 2.434	1.328	0.258	0.041
T2	**4.58 ± 2.644**	**2.23 ± 2.127**	**7.969**	0.010*	**0.257**
FACIT-sp					
T0	25.00 ± 10.892	24.84 ± 9.376	–	–	–
T1	23.13 ± 12.371	27.53 ± 8.322	3.207	0.083	0.091
T2	27.00 ± 12.364	32.29 ± 7.819	2.683	0.113	0.090
ECR-Avoidant					
T0	27.88 ± 6.051	29.00 ± 6.280	–	–	–
T1	29.88 ± 7.527	28.11 ± 5.415	1.383	0.248	0.041
T2	27.00 ± 12.364	32.29 ± 7.819	**4.407**	0.044*	**0.128**
ECR-Anxiety					
T0	26.71 ± 10.999	31.26 ± 8.366	–	–	–
T1	27.13 ± 11.407	28.42 ± 12.025	0.017	0.896	0.001
T2	24.80 ± 11.912	25.24 ± 11.206	0.038	0.847	0.001
QUAL-EC-Relationship					
T0	17.65 ± 3.605	16.63 ± 3.715	–	–	–
T1	17.44 ± 3.881	15.26 ± 4.026	2.298	0.139	0.067
T2	16.00 ± 4.408	17.29 ± 4.883	0.695	0.411	0.023
QUAL-EC- Preparation					
T0	13.82 ± 3.107	14.11 ± 2.885	–	–	–
T1	14.38 ± 4.515	13.16 ± 4.086	0.948	0.337	0.029
T2	13.13 ± 3.114	13.29 ± 3.236	0.116	0.736	0.004
QUAL-EC- Life Completion					
T0	18.06 ± 4.630	17.68 ± 3.449	–	–	–
T1	17.31 ± 3.877	17.63 ± 3.022	0.134	0.717	0.004
T2	16.13 ± 3.852	18.71 ± 3.098	**5.493**	0.026*	**0.155**

***p < 0.001; **p < 0.01; *p < 0.05.

Partial η^2^: 0.01 small effect size, 0.06 moderate effect size, 0.14 large effect size.

Significant values are in bold.

**Table 4:** T-test results of changing range in primary and secondary outcomes.

	WLC Group(n = 17) M ± SD	CALM Group (n = 19) M ± SD	*F*	*sig*	*t*	*p*	Cohen’s d	95% confidence interval	
								Lower	Upper
Primary outcomes									
ΔDADDS (T0-T1)	2.44 ± 21.420	10.16 ± 13.263	4.565	0.040	− 1.254	0.201	− 0.442	− 1.113	− 0.234
ΔDADDS (T0-T2)	5.40 ± 24.994	13.28 ± 14.652	2.838	0.102	− 1.127	0.268	− 0.432	− 1.087	0.301
Secondary outcomes									
ΔPHQ-9 (T0-T1)	1.38 ± 8.049	4.47 ± 6.695	0.433	0.515	− 1.244	0.222	− 0.422	− 1.092	0.254
ΔPHQ-9 (T0-T2)	0 ± 5.843	6.44 ± 7.587	0.387	0.539	− 2.689	0.011*	− 0.940	− 1.657	− 0.210
ΔDT (T0-T1)	0.75 ± 3.416	2.11 ± 3.197	0.017	0.896	− 1.200	0.239	− 0.412	− 1.067	− 0.248
ΔDT (T0-T2)	− 0.67 ± 1.723	1.79 ± 3.068	2.230	0.148	− 2.453	0.022*	− 0.965	− 1.773	− 0.139
ΔFACIT-sp (T0-T1)	− 2.31 ± 7.863	2.68 ± 8.938	0.338	0.565	− 1.739	0.091	− 0.590	− 1.266	0.094
ΔFACIT-sp (T0-T2)	1.87 ± 9.303	6.40 ± 8.998	0.824	0.372	− 1.357	0.186	− 0.495	− 1.218	0.236
ΔECR-Avoidance (T0-T1)	− 2.06 ± 4.892	0.89 ± 7.752	3.452	0.072	− 1.319	0.196	− 0.448	− 1.118	0.224
ΔECR-Avoidance (T0-T2)	− 3.67 ± 4.353	0.56 ± 6.428	3.422	0.074	− 2.162	0.038*	− 0.756	− 1.460	− 0.040
ΔECR-Anxiety (T0-T1)	− 0.44 ± 13.604	2.84 ± 11.644	0.285	0.597	− 0.769	0.448	− 0.261	− 0.927	0.409
ΔECR-Anxiety(T0-T2)	2.27 ± 14.921	5.89 ± 9.707	0.591	0.448	− 0.840	0.407	− 0.294	− 0.980	0.398
ΔQUAL-Relationship (T0-T1)	0.19 ± 4.996	1.37 ± 5.336	0.049	0.826	− 0.671	0.507	− 0.228	− 0.893	0.441
ΔQUAL-Relationship (T0-T2)	1.20 ± 5.171	− 0.50 ± 4.902	0.002	0.964	0.968	0.341	0.338	− 0.355	1.026
ΔQUAL-Preparation (T0-T1)	0.69 ± 5.375	− 0.95 ± 3.597	1.078	0.307	1.072	0.291	0.364	− 0.310	1.032
ΔQUAL-Preparation (T0-T2)	− 0.67 ± 4.100	− 0.56 ± 3.823	0.149	0.703	− 0.080	0.936	− 0.028	− 0.713	0.657
ΔQUAL-Completion (T0-T1)	− 0.63 ± 4.288	− 0.05 ± 4.156	0.058	0.811	− 1.784	0.692	− 0.136	− 0.801	0.531
ΔQUAL-Completion (T0-T2)	− 1.60 ± 3.888	1.33 ± 5.280	1.767	0.193	− 1.717	0.084	− 0.624	− 1.321	0.083

Δ: the changing of measurements indicators in T1 and T2, compared to T0;

***p < 0.001; **p < 0.01; *p < 0.05;

Small effect size: 0 < Cohen’s d ≤ 0.2; Moderate effect size: 0.2 < Cohen’s d ≤ 0.8; large effect size: Cohen’s d > 0.8

### Secondary outcomes

A comparison of various psychometric scores for the CALM group and the WLC group is presented in Table 2. No difference was found in T0 and T1 results for the two groups. For the T2-conducted after six sessions, the percentage of participants experiencing suicide ideation in the CALM group in significantly lower than that in the WLC group (23.5% vs. 60.0%, χ^2^ = 4.394, *p* = 0.036); Lower distress scores were recorded in the CALM group than that in the WLC group (t = 2.460, *p* = 0.022); and the scores on Life completion was significantly better in the CALM group than that in the WLC group (t = − 2.093, *p* = 0.045).

For the secondary outcomes analysed using ANCOVA, the participants in the CALM group had significantly lower levels of depression than those in the WLC group (F = 5.016, *p* = 0.033) at T2, after adjusting for baseline PHQ-9 scores. Partial η2 = 0.143 indicated a large effect size; in addition, participants in the CALM group reported significantly lower levels of distress (F = 7.969, *p* = 0.010), as reflected in their DT scores, and attachment avoidance (F = 4.407, *p* = 0.044), as reflected in their ECR-avoidance scores, than participants in the WLC group. Furthermore, participants in the CALM group reported a significantly higher sense of life completion (F = 5.493, *p* = 0.026), as reflected in their QUAL-EC-Life Completion scores, than participants in the WLC group, with the effect size ranging from moderate (partial η2 = 0.128 in ECR-avoidance) to large (partial η2 = 0.257 in DT). No significant benefits were found for any of the outcomes in T1 analysed using ANCOVA. We explored the differences between the two groups in the changes in scores on the secondary psychometric indicators before and after the interventions (Table 4). The results showed that the decline in depression and distress score between T0 and T2 was more remarkable in the CALM group than in the WLC group (PHQ-9: T0-T2, t = − 2.689, *p* = 0.011, Cohen’s d = 0.94; DT: T0-T2, t = − 2.453, *p* = 0.022. Cohen’s d = 0.965).

### Acceptability of CALM therapy in Chinese patients with metastatic breast cancer

The results presented in Table 5 show that study participants felt that the CALM therapy had overall positive effects on helping them to cope with stage IV breast cancer. The percentage of participants scoring ≥ 2 and ≥ 3 on the CEQ-2 increased on all the 13 items from T1 to T2. At T1 (conducted after three CALM therapy sessions), the percentage of participants scoring ≥ 2 on the CEQ-2 ranged from 57.9% on item 7 to 100% on item2 ; at T2, it ranged from 88.9% on item 6 to 100% on item 1, 2, 3, 5, 6, and 12. The participants reported lower levels of benefit from CALM therapy on items related to death and dying (Items 7–9) than on other items; However, at the T2 time point, the scores on these three death/dying-related items were apparently comparatively higher than at T1.

**Table 5:** Results from clinical evaluation questionnaire-2.

Items: to what extent has CALM therapy helped you to: (0 = Not at all; 1 = A little bit; 2 = Somewhat; 3 = Quite a bit; 4 = Very much)	Percentage of scoring ≥ 2		Percentage of scoring ≥ 3	
	T1	T2	T1	T2
1. Freely discuss my concerns about cancer and my treatment options	18/19 (94.7)	18/18 (100)	10/19 (52.6)	13/18 (72.2)
2. Talk and feel understood about how cancer has affected my life	19/19 (100)	18/18 (100)	13/19 (68.4)	13/18 (72.2)
3. Deal with changes in my relationships as a result of cancer	18/19 (94.7)	18/18 (100)	9/19 (47.4)	10/18 (55.6)
4. Explore better ways to communicate with my health care team, my family and others	15/19 (78.9)	17/18 (94.4)	10/19 (52.6)	16/18 (88.9)
5. Clarify my values and beliefs	18/19 (94.7)	18/18 (100)	11/19 (57.9)	11/18 (61.1)
6. Talk about my concerns about the future and to be less frightened	18/19 (94.7)	18/18 (100)	12/19 (63.2)	12/18 (66.7)
7. Plan and prepare for what can happen at the end of life	11/19 (57.9)	16/18 (88.9)	6/19 (31.6)	9/18 (50.0)
8. Express and manage my fears about dying	13/19 (68.4)	17/18 (94.4)	8/19 (42.1)	9/18 (50.0)
9. Talk about what will happen to my family	15/19 (78.9)	14/18 (77.8)	8/19 (42.1)	11/18 (61.1)
10. Say important things to my loved ones	16/19 (84.2)	16/18 (88.9)	9/19 (47.4)	12/18 (66.7)
11. Make the most of my time	16/19 (84.2)	17/18 (94.4)	11/19 (57.9)	13/18 (72.2)
12. Enjoy and live in the present	18/19 (94.7)	18/18 (100)	12/19 (63.2)	17/18 (94.4)
13. Live with an uncertain future	17/19 (89.5)	17/18 (94.4)	10/19 (52.6)	15/18 (83.3)

## Discussion

This pilot trial examined the feasibility of CALM therapy for patients with metastatic breast cancer and significant death anxiety. The study participants were randomly assigned to one of two groups using computer-implemented dynamic randomization, thus achieving a relatively even distribution of demographic and sociological variables between the two groups. Our hypothesis was that in a sufficiently large study sample size, CALM therapy would significantly alleviate psychological burden and improve quality of life in patients with metastatic breast cancer. Although the differences in death anxiety at matched time points post-intervention did not achieve significance in this small pilot randomized controlled trial, we did observe significant between-group differences in several secondary outcome measures, with the patients benefitting significantly from receiving full six sessions of CALM therapy on the following metrics: depression severity, distress level, attachment avoidance and sense of life completion, with the effect size ranging from moderate to large. The percentage of patients with suicide ideation also decreased more in the CALM group than in the WLC group. Furthermore, the progressive differences in the levels of depression and death anxiety between the two groups shows and apparent and a gradual trend. Further exploration of changes in scores gave us more positive indications of psychological improvement in depression, distress, and attachment avoidance resulting from the CALM therapy, with a large effect size. Based on the results presented in Table 5, it can be seen that the study participants exhibited a high degree of acceptance towards CALM therapy. The largest shifts between T1 and T2 are evident in the psychometric items related to enjoying the present, living with uncertainty, making the most of time, communicating with others and discussing future concerns, which indicates that CALM therapy may have a particular efficacy around psychosocial outcomes related to discovering meaning in life and peace in the face of stage IV breast cancer. A discussion about death and dying was open after three sessions if it fit the patients' preferfences, and these discussions were generally introduced through two opportunities: talking about their future and the uncontrolled aspects of living with advanced cancer life. Significant death anxiety in Chinese advanced cancer patients has been reported by researchers^31–33^, and talking about death and dying with this population during our research was not as difficult as reported in a previous study^34^. However, the magnitude of improvements in the domains of ‘planning end-of-life’ and ‘fears of death and dying’ was small, as not all participants were willing to enter into a discussion of death and dying. This indicates that our online CALM therapy content needs to be enhanced in this area in the future researches.

This was the first trial of online CALM therapy for patients with metastatic breast cancer in China. The online delivery model ensured that the intervention and research proceeded at intended pace. Even outside periods of COVID-19 community lockdowns, the patients were still reluctant to visit cancer hospitals-which can be distressful environments that reinforce their cancer patients identity- except when required for anti-cancer treatments and examinations. Limitations of online CALM therapy also existed. Bertuzzi et al. reported common issues, including challenges with technology, low user motivation, and privacy/safety concerns^35^. We also encountered challenges with technology, as we could not provide non-verbal empathetic response to patients’ strong emotional expressions, such like crying. However, the low motivation and privacy/safety were not remarkable in our study, as most patients agreed to participate, knowing it would be conducted online and hospital visits were not required. The widespread everyday use of WeChat video calls in China may also provide some reassurance about the privacy/safety.

Patients in both groups demonstrated good compliance, with over 94% completing all six CALM sessions and the three assessments. This high compliance rate indicates that CALM therapy was well received by the study population. Compliance in interventional clinical researches can be influenced by inclusion criteria, recruiting procedures and psychotherapy relationship. Published study protocols for randomized clinical trials of CALM therapy have used different inclusion criteria across various study populations. Rodin’s phase III RCT and Miyamoto’s phase 2 trial protocol did not set the threshold scores in the main outcomes^36^; while Caruso required baseline score ≥ 10 on the Patient Health Questionnaire (PHQ-9) and/or ≥ 20 on the Death and Dying Distress Scale (DADDS)^23^; Scheffold et al. also limited the inclusion with ≥ 9 on the Patient Health Questionnaire (PHQ-9) and/or ≥ 5 on the Distress Thermometer (DT)^37^. CALM researchers had not yet agreed on this issue. Our pre-testing revealed that removing threshold score increased recruiting ratio, but decrease the full study compliance in this population in China. In our study, after weighing the intervention impact and population accessibility, we set a DADDS ≥ 15 as inclusion threshold. This was based on the notion that patients with significant death anxiety, who have a considerable need for psychotherapy, may demonstrate positive compliance in clinical trials-in which CALM therapy can serve them well. In addition, engaging breast cancer nurses in the same department from which the patients were recruited facilitated the recruiting process and likely improved the study participants’ compliance.

In the CALM therapy, engaging family members in the intervention process is encouraged. Throughout our pre-experiment and during this pilot trial, we made consistent efforts to request participation from the husbands of patients or their other family members. However, this request was refused in the vast majority of cases. The patients’ husbands believed that only the patients themselves needed psychological therapy, as they did not feel they had any problems that needed addressing. In the context of Chinese culture, men are accustomed to taking on the role of decision-maker rather than caregivers. Even when their wives were suffering from metastatic breast cancer, the husbands were more inclined to participate in deciding on the anti-cancer treatment plans while seldom providing psychological support. Patients also refused our requests to engage their parents or children-whom they were trying to protect. Patients were reluctant to let these family members witness their psychological distress in addition to their physical distress. The reluctance of husbands to participate in therapy sessions and this shielding of vulnerable family members highlight critical cultural barriers and misconceptions about mental health and psychological support. Enhanced education efforts and outreach to destigmatize therapy and elucidate its offerings may enable improved family engagement in the future.

Previous CALM studies in China focused on cancer survivors, examining the effects of CALM therapy on cognitive impairment and immunity markers^38^. However, the main advantages of CALM therapy are the benefit of psychological, and it improves the overall quality of life for patients with advanced, life-threatening illnesses confronting the finitude of life. All the CALM researches in other countries have examined CALM therapy from this perspective^23,39,40^. In this study, with a small sample, we verified psychosocial benefits of CALM therapy for Chinese patients with metastatic breast cancer. The encouraging results in this pilot study lay groundwork for an adequately-powered phase III RCT of CALM therapy for Chinese patients with metastatic breast cancer.

## Limitations

This first exploration of CALM therapy in Chinese patients with metastatic breast cancer provids encouraging support for a larger phase III RCT to be conducted in this population in China. However, there were several limitations to the study. For instance, because the small sample is small, the results cannot indubitably verify the effects of CALM therapy on depression and death anxiety in patients with metastatic breast cancer. Because we recruited patients from only one tertiary center in Beijing who have generally high levels of education, generalizability is limited, and the results of the study may not reflect outcomes representative of the broader population in China. Although online delivery aided intervention accessibility and pace, there was a challenge with technology: the inability to provide face-to-face empathic support. However, these shortcomings hardly negate the value of our online approach in demonstrating feasibility of CALM therapy and providing support for utilising this approach in the future studies. Indeed, the limitations of this study help to inform such efforts. With only one dropout per arm, compliance was adequate, indicating the acceptability of both study procedures and the CALM therapy. Thus, these positive indicators favor launching a multi-centered phase III study with a large sample size to rigorously evaluate the effects of CALM therapy in the future.

## Conclusions

This pilot randomized controlled trial provides preliminary evidence supporting the efficacy of CALM therapy for alleviating depression, distress, death anxiety, suicide ideation in Chinese patients with metastatic breast cancer. Furthermore, CALM therapy has the potential to enhance patients’ sense of life completion at the end of life. A low dropout rate and high intervention compliance indicate that CALM therapy is a welcome and feasible individual psychotherapy for this population in China. These promising findings warrant further rigorous examination in a phase III randomized controlled trial with a large Chinese metastatic breast cancer cohort. The findings also reveal that specially trained therapists can effectively administer online CALM to patients with metastatic breast cancer, which can help ensure intervention accessibility and continuity amidst healthcare disruptions and patients’ reluctance to visit the hospital.

## Data Availability

Data supporting the findings of this study and supplementary material are available from the corresponding author upon reasonable request.
